# Is the incidence of sandwich vertebral fracture higher than that of ordinary adjacent vertebral fracture after PKP?

**DOI:** 10.1097/MD.0000000000029900

**Published:** 2022-07-08

**Authors:** Bo Yang, Yu Zhao, Yangxue Zhao

**Affiliations:** a Graduate School of Xi’an Medical University, Xi’an, China; b Department of Orthopaedics, The Ninth Hospital of Xi’an, Xi’an, China.

**Keywords:** osteoporotic vertebral compression fracture, sandwich vertebrae, percutaneous kyphoplasty

## Abstract

**Objective:**

To compare the incidence of fracture between sandwich vertebra and ordinary adjacent vertebra after percutaneous kyphoplasty (PKP).

**Method:**

We analyzed 225 consecutive patients with osteoporotic vertebral compression fractures who underwent PKP between January 2016 and December 2020 at our medical institution. The sandwich vertebrae was located between 2 cement-augmented vertebra and was followed for at least 12 months. The clinical data of patients with sandwich vertebra and ordinary adjacent vertebra were recorded, and the incidence of postoperative fracture between sandwich vertebra and ordinary adjacent vertebra was compared.

**Results:**

The mean continuous follow-up time was 31.30 ± 18.04 months in patients with sandwich vertebra and 25.85 ± 7.96 months in patients with ordinary adjacent vertebra. It should be noted that the incidence of sandwich vertebral fractures was 10.00%, which was not statistically higher than 3.26% for ordinary adjacent vertebral fractures. However, a significant difference was observed in the cement volume of single vertebral body, procedure time, and bleeding.

**Conclusion:**

Although the volume of cement in a single vertebral body is less and the procedure time and bleeding are more, the incidence of sandwich vertebral fracture is not higher than that of ordinary adjacent vertebral body.

## 1. Introduction

As hypertension, diabetes, coronary heart disease, and so on, osteoporosis has gradually become a serious risk factor affecting the health of the global population. An estimated 200 million people worldwide suffer from osteoporosis.^[1]^ China, a country with about 19% of the world’s population, has even more osteoporosis sufferers.^[2]^ It is predicted that by the middle of this century, the number of osteoporosis patients in China will reach 400 million, which will seriously hinder the healthy development of the population of China and the world.^[3]^ Due to the decrease of bone mass and destruction of bone tissue structure in patients with osteoporosis, the susceptibility to fracture is significantly higher than that in the general population. This has been followed by vertebral compression fractures, which have been devastating to older people around the world, especially postmenopausal women.^[4]^

Immobilization is a high-risk factor for respiratory and urinary tract infections, but for osteoporotic vertebral compression fractures (OVCFs), a number of expert associations recommend minimally invasive surgery to avoid long-term immobilization and lower patient life expectancy.^[5]^ Percutaneous vertebral augmentation, as the most commonly used minimally invasive surgery, can quickly reduce the pain and restore the activity function of patients with OVCFs, which is recognized and praised by the majority of orthopedic doctors.^[6–9]^ However, some patients who received cement-augmented vertebra experienced a recurrence of vertebral fractures, and most of the new fractures occurred in the vicinity of the previous cement-augmented vertebra.^[10]^ The incidence of adjacent vertebral fractures has been reported to be as high as 52%, which is a great pain for patients and a heavy financial burden for families.^[11,12]^

Multiple percutaneous vertebral augmentation can form sandwich vertebrae, which is a well-preserved vertebral body between 2 cement-augmented vertebra. Some scholars believe that the hardness and stiffness of the vertebral body augmented with cement increase, which enlarges the load of the adjacent vertebral body and leads to fracture.^[13]^ Therefore, the sandwich vertebrae, a special vertebrae, receives the double load transmission from the upper and lower vertebra augmented with cement, so that the probability of fracture is greater than that of ordinary adjacent vertebra in theory. We found that there are few studies on the incidence of sandwich vertebral fractures, the most important of which is that there are several types of minimally invasive procedures, such as percutaneous vertebroplasty (PVP) and PKP, which may lead to errors in accuracy. And few studies have focused solely on post-PKP fractures of the sandwich vertebrae.

Therefore, we put forward the conjecture whether the incidence of sandwich vertebral fracture after PKP is significantly higher than that of ordinary adjacent vertebral fracture. So, we retrospectively conducted a continuous study on eligible patients in our medical institution to determine the incidence of sandwich vertebral fracture and ordinary adjacent vertebral fracture, so as to make up for this research gap.

## 2. Materials and Methods

### 2.1. Study design

Between January 2016 and December 2020, a total of 317 patients underwent PKP, a minimally invasive surgical procedure, for osteoporotic vertebral compression fractures at our medical institution. Therefore, we conducted a well-designed retrospective study of these patients. The main conclusions of this study were obtained by comparing the incidence of postoperative fractures between sandwich vertebra and ordinary adjacent vertebra. Of course, we also compared some parameters often recorded by the clinician.

### 2.2. Study population

Although 317 OVCFs patients were initially recorded successfully completing PKP treatment, participants in this study had to meet the following inclusion criteria: (1) Osteoporotic vertebral compression fracture was mainly diagnosed before operation; (2) fresh fractures, that is, within a month; (3) it is consistent with the diagnosis of sandwich vertebral body, or ordinary adjacent vertebral body produced by single segment surgical vertebrae; (4) patients were able to cooperate successfully with the study; (5) continuous follow-up lasted at least 12 months. Ineligible patients are due to meeting the following criteria: (1) old fracture, that is, the fracture occurred more than 1 month; (2) a multisegmental vertebral fracture that does not form a sandwich vertebrae; (3) nerve damage due to compression of the spinal canal; (4) patients with disorders of consciousness, such as Alzheimer disease, can not cooperate with the study; and (5) follow-up data were not available.

### 2.3. Percutaneous kyphoplasty: technical considerations

All operations were performed under the guidance of C-arm fluoroscopy via pedicle approach under local anesthesia. The patient was placed on the operating table in prone position. Local anesthesia (2% lidocaine, 1% ropivacaine, and saline 1:2:3) was administered. The 11-gauge needle was then inserted into the anterior one-third part of the vertebral body slowly along the pedicle of vertebrae under the guidance of C-arm fluoroscopy. A dilatable balloon injected with contrast medium is placed in the anterior portion of the vertebral body and carefully expanded to restore the satisfactory height of the compressed vertebral endplate. After the above basic operation is carried out, the cement of the drawing stage is gradually pushed into the vertebral body. Each one-fourth tube of cement is injected and stopped to see if the cement is leaking. The operation was completed after satisfactory filling of bone cement in the vertebral body of the fracture. It should be noted that the operation was stopped as soon as cement spread to the posterior wall of the vertebral body.

### 2.4. Postoperative treatments

After returning to the ward, patients were continuously monitored by ECG for 6 hours. at the same time check blood routine, renal function, if no abnormal indicators were revealed then for postoperative antiosteoporosis treatment, such as bisphosphonate, calcitriol. The second day after operation, recheck the image data, such as X-ray film and CT. If there is no abnormal image performance, patients can wear the branch to get out of bed or end hospitalization.

### 2.5. Study data collection

The preoperative data of all eligible patients were collected retrospectively. General indexes such as age, sex, fracture position, body mass index (BMI), volume of cement injected into a single vertebral body, volume of intraoperative bleeding, and operation time were recorded.

We used Visual Analog Scale (VAS) score and Oswestry Disability Index (ODI), the most commonly used in the study, to record the pain symptoms and activity function of patients. The former means that higher the score, the more unbearable the pain. And the latter means that the higher the score, the more unable daily activities are to be carried out.

The most important is the continuous observation of sandwich vertebra and ordinary adjacent vertebra. Once the patient has a complaint of lumbar and back pain during follow-up, an immediate MRI examination of the spine is performed to detect the first time whether a fracture has occurred again.

### 2.6. Statistical analysis

All statistical analysis was carried out in SPSS 18.0. Categorical data such as sex, incidence of fractures were analyzed using chi-square test and continuous data such as age, BMI, VAS, and ODI were compared using Student t-test. The statistically significant difference was identified where *P* < .05 with hypothesis testing using a 2-tailed test of significance.

## 3. Results

### 3.1. Object characteristics

**Figure 1.**
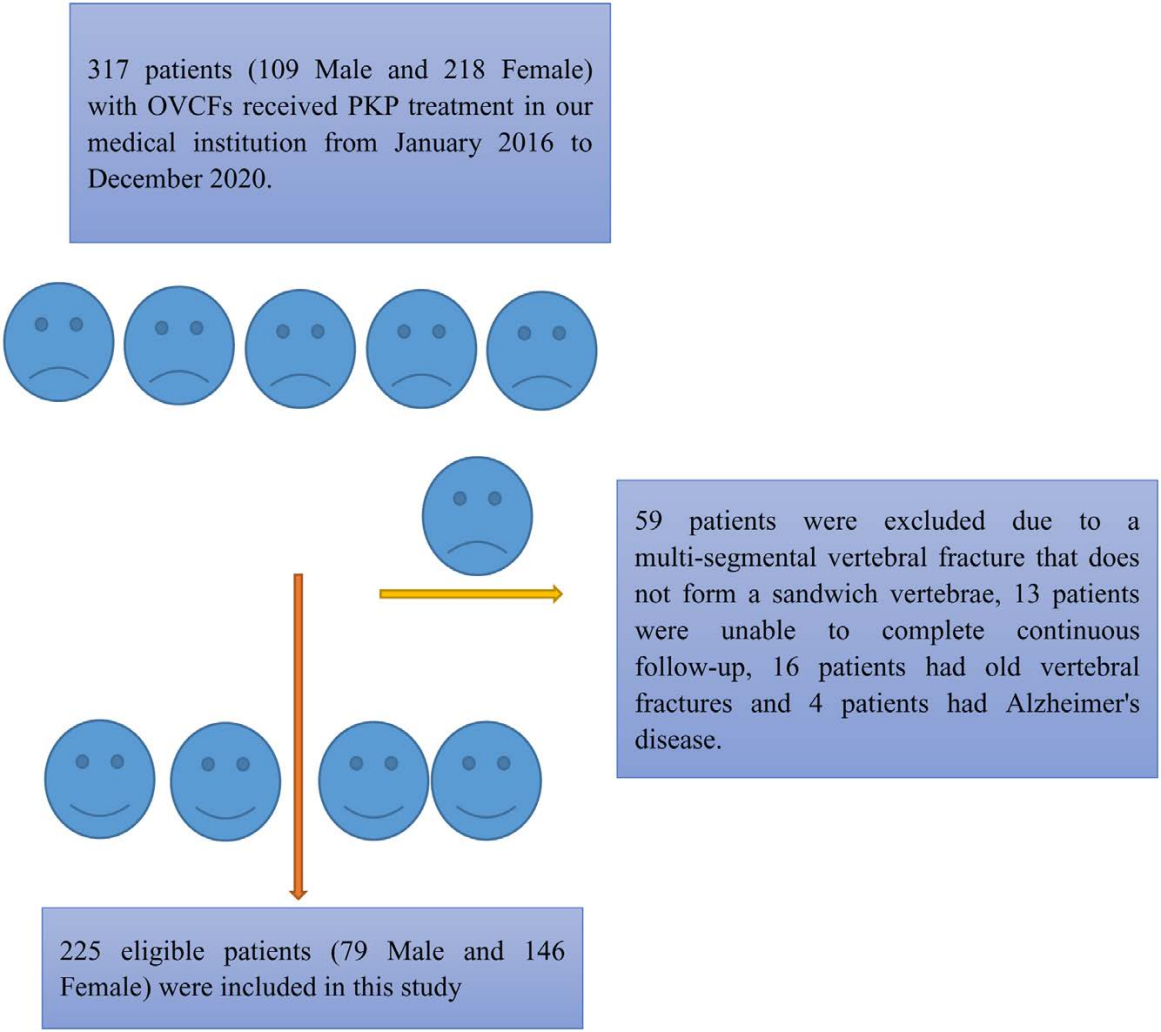
Research subjects.

**Figure 2.**
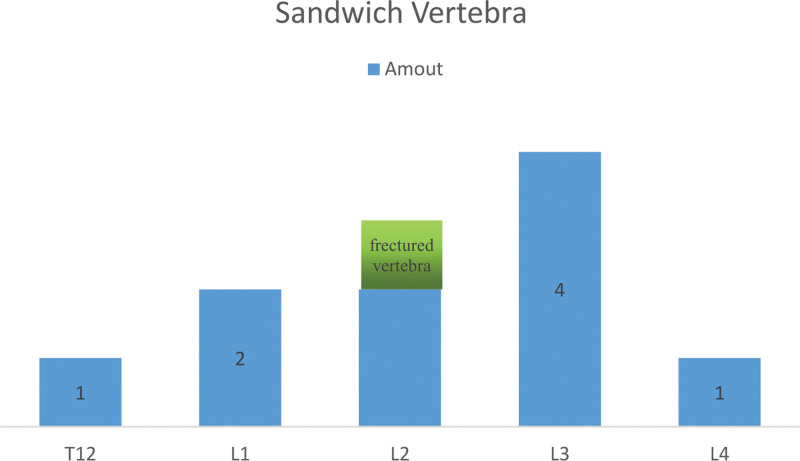
Sandwich vertebra. The sandwich vertebrae are located in the thoracolumbar junction area. There were 1 case of T12, 2 cases of L1, 2 cases of L2, 4 cases of L3, and 1 cases of L4. An 85-year-old woman with osteoporosis suffered a fracture of her sandwich vertebra (L2) 1 month after receiving PKP treatment.

Three hundred seventeen patients (109 male and 218 female) with OVCFs received PKP treatment in our medical institution from January 2016 to December 2020. However, 59 patients were excluded due to a multisegmental vertebral fracture that does not form a sandwich vertebrae, 13 patients were unable to complete continuous follow-up, 16 patients had old vertebral fractures, and 4 patients had Alzheimer disease. Finally, only 225 eligible patients (79 male and 146 female) were included in this study, 10 patients (1 male and 9 female) had sandwich vertebra, and the remaining 215 patients (87 male and 128 female) were ordinary adjacent vertebra owners (Fig. 1). Patients with sandwich vertebra and ordinary adjacent vertebra were followed up for an average of 31.30 ± 18.04 and 25.85 ± 7.96 months, respectively, with no significant difference identified. The sandwich vertebrae are located in the thoracolumbar junction area. There were 1 case of T12, 2 case of L1, 2 case of L2, 4 case of L3, and 1 cases of L4 (Fig. 2). The mean age of patients with sandwich vertebra was 73.80 ± 10.94, which was not significantly different from that of patients with normal adjacent vertebra at 75.36 ± 8.45. There was no significant difference in other basic data, such as gender, fracture position, BMI, preoperative VAS, and ODI. However, it is interesting to note that differences in the volume of cement injected into a single vertebral body, the volume of bleeding, and the duration of surgery were revealed. The above information is detailed in Table 1.

**Table 1:** Basic demographic data of participants

	Age	Sex (Female:male)	Position (T:L)	BMI	Volume	Bleeding	Time	Follow-up (M)	Pre-VAS	Pre-ODI
Group S (sandwich vertebra)	73.80 ± 10.94	5:5	1:9	22.27 ± 2.63	3.57 ± 0.69	38.00 ± 15.85	62.00 ± 4.22	31.30 ± 18.04	7.80 ± 0.63	74.80 ± 4.34
Group A (adjacent vertebra)	75.36 ± 8.45	141:74	87:127	22.90 ± 2.60	5.15 ± 1.00	17.72 ± 7.44	46.09 ± 7.68	25.85 ± 7.96	7.47 ± 0.80	72.40 ± 4.73
*P*	.573	.327	.093	.459	.000	.000	.000	.366	.191	.118

There was no significant difference in age, gender, fracture position, BMI, Follow-up time, preoperative VAS and ODI. However, it is interesting to note that differences in the volume of cement injected into a single vertebral body, the volume of bleeding, and the duration of surgery were revealed.

BMI = body mass index, ODI = Oswestry Disability Index, VAS = Visual Analog Scale.

### 3.2. Clinical outcomes

VAS score and ODI are frequently used in the study. We retrospectively collected the VAS pain score and ODI score of all subjects. The average VAS score of sandwich patients was 2.60 ± 0.70 and that of ordinary adjacent vertebral patients was 3.09 ± 1.08. No difference was discovered. But significantly less than preoperative scores. The same results were obtained when ODI score were collected from all patients after operation. The postoperative ODI scores of sandwich vertebral patients and ordinary adjacent vertebral patients were 28.30 ± 6.80 and 30.14 ± 3.40, respectively. There was no difference, but they were significantly less than the preoperative scores (Table 2).

**Table 2:** Clinic outcomes

	Post-VAS	Post-ODI
Group S (sandwich vertebra)	2.60 ± 0.70	28.30 ± 6.80
Group A (adjacent vertebra)	3.09 ± 1.08	30.14 ± 3.40
*P*	.155	.417
*P* (compared with preoperative)	.000	.000

There was no difference, but they were significantly less than the preoperative scores.

ODI = Oswestry Disability Index, VAS = Visual Analog Scale.

### 3.3. Sandwich vertebra and ordinary adjacent vertebra

**Figure 3.**
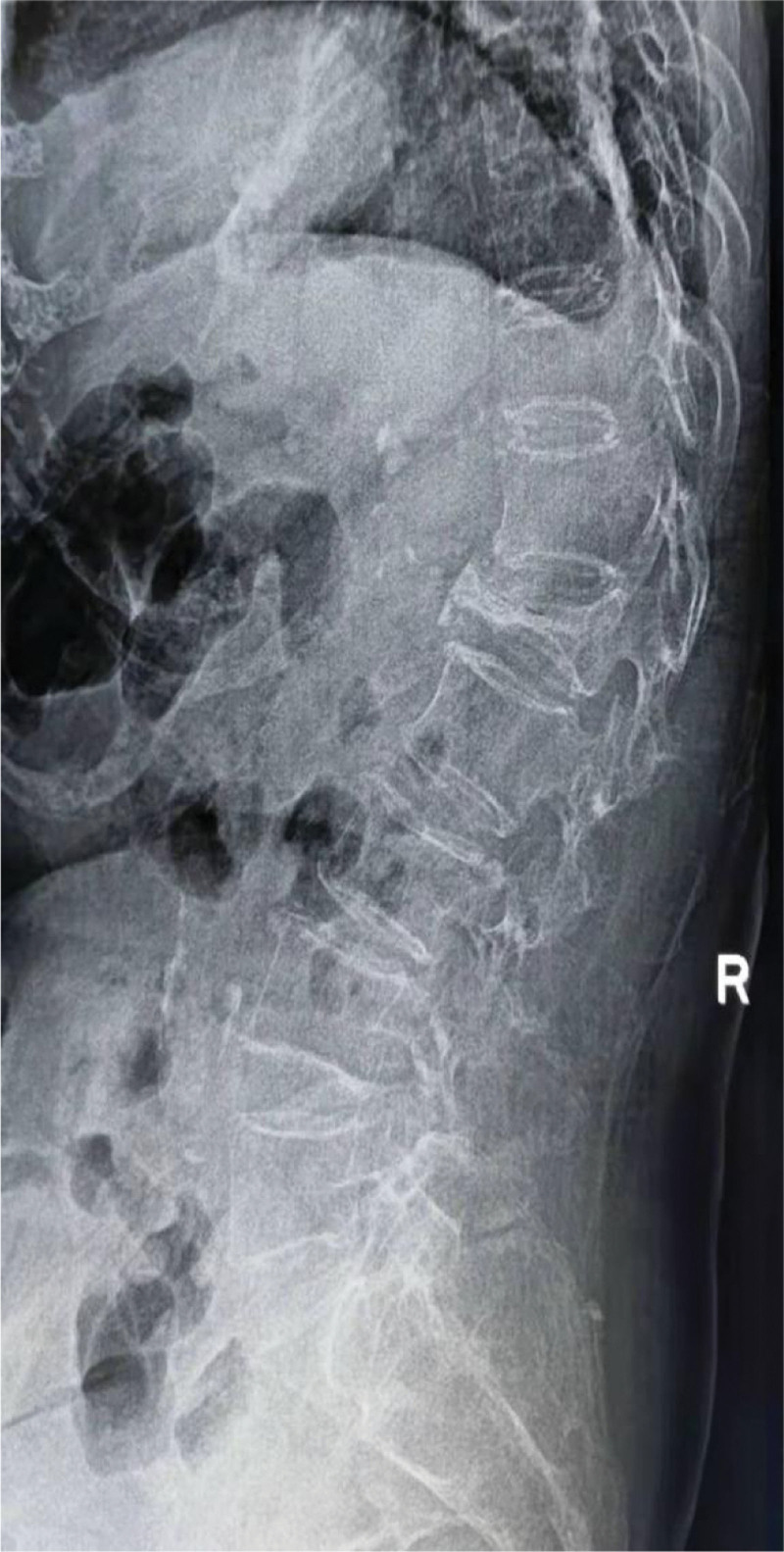
(A) An 85-year-old woman with osteoporosis presented with lumbar pain for 2 days. DR revealed compression fractures of the L1 and L3. (B) T1-weighted image indicated low signal intensity within L1 and L3. (C) T2-STIR image indicated high signal intensity within L1 and L3. (E) PKP was implemented in L1 and L3 in our medical institution, and L2, a sandwich vertebrae was also formed, as shown in (D) and (E). (G) One month after the initial treatment, the patient again sought medical attention due to low back pain. T1-weighted image manifested low signal intensity and T2-STIR image indicated high signal intensity in L2, a sandwich vertebral body, As shown in (F) and (G).

One of the 10 patients with sandwich vertebra complained of low back pain during an average follow-up of 31.30 ± 18.04 months. An MRI of the spine immediately revealed the presence of a sandwich vertebral L2 fracture, as shown in Figure 3. For patients with ordinary adjacent vertebra, 7 patients also had complaints of low back pain during a continuous follow-up of 25.85 ± 7.96 months. Inevitably, MRI showed that 7 patients were proved to have ordinary adjacent vertebral fractures. The incidence of sandwich vertebral fracture was 10.00%, which was higher than 3.26% of ordinary adjacent vertebral body, but no difference was transmitted after careful statistical comparison (Table 3).

**Table 3:** Subsequent vertebral fracture

	Fracture	Well	
Group S (sandwich vertebra)	1	9	
Group A (adjacent vertebra)	7	208	
Total	8	217	
*P*			.309

No difference was transmitted.

## 4. Discussion

Bending over to pick up things and turning over in bed in daily life can lead to fractures in patients with osteoporosis to a great extent. According to statistics, there are 1000 brittle fractures caused by osteoporosis every second in the world, of which about half occur in the vertebral body.^[14]^ It is a frequent occurrence in China, one of the world’s Cradle of civilization. It has been reported in Hong Kong that about 30% of the elderly have osteoporotic fragile fractures.^[15]^ Since minimally invasive surgery was first reported in 1987, infusions of cement, a bone adhesive, into fractured vertebrae have resulted in effective treatment for the vast majority of patients with OVCFs.^[16]^ Since the beginning of this century, PKP has been one of the most commonly used minimally invasive procedures for the treatment of osteoporotic vertebral compression fractures due to its advantages of minimal trauma and rapid improvement of symptoms.^[17]^ Therefore, all patients in our medical institution are treated with PKP.

When basic data were collected, it was suggestive that the volume of cement in a single vertebral body is less and the procedure time and bleeding are more in patients with sandwich vertebra. Only one vertebral body in all patients with ordinary adjacent vertebra received cement augmentation; however, patients with sandwich vertebra had at least 2 fractured vertebrae that had received cement augmentation. Therefore, we injected cement bilaterally through the pedicle for a single fractured vertebral body, and unilaterally through the pedicle for 2 or more fractured vertebral bodies. As a result, the volume of cement in a single vertebral body in sandwich vertebrae was smaller than that in ordinary adjacent vertebral body patients. It is consistent with the conclusion of several meta-analyses that the volume of cement used in unilateral Percutaneous kyphoplasty is less than that of conventional bilateral percutaneous kyphoplasty.^[18,19]^ The operation time of unilateral percutaneous kyphoplasty is less than that of bilateral percutaneous kyphoplasty, but the operation time of unilateral PKP for 2 or more fractured vertebral bodies is significantly higher than that of bilateral PKP for 1 vertebral body. The number of fractured vertebral bodies in sandwich vertebral body patients is more than that in ordinary adjacent vertebral body patients, resulting in an increase in the probability of provoking paravertebral and internal vertebral vessels during operation, and a significant increase in the amount of bleeding. These differences, however, did not affect our findings.

We performed a retrospective analysis of all patients treated with PKP, including significant clinical data, because the patients who came to our medical institution for help were prompted by low back pain. However, this important data were not recorded in the recent study of Ping-Yeh et al.^[20]^ As we recorded the preoperative data, although the number of vertebral fractures was high in the sandwich vertebral bodies, the preoperative average VAS score of the 10 patients with the sandwich vertebral bodies was 7.80 ± 0.63, with no difference from the preoperative average of 7.47 ± 0.80 in the ordinary adjacent vertebral bodies. After the minimally invasive procedure, all patients reported reduced pain. The postoperative VAS score of 10 patients with sandwich vertebral bodies was 2.60 ± 0.70 and that of ordinary adjacent vertebral bodies was 3.09 ± 1.08. Although there was no identified difference between the 2 groups, they were statistically less than that before operation. The same findings were identified when the ODI score was collated. There was no significant difference between the preoperative ODI score of 74.80 ± 4.34 in the 10 patients with sandwich vertebra and 72.40 ± 4.73 in patients with the ordinary adjacent vertebra. Patients who received PKP showed significant improvement in mobility because of the rapid relief of postoperative low back pain. There was no detectable difference in the ODI score, the postoperative ODI score of 10 patients with sandwich vertebral bodies was 28.30 ± 6.80 and that of ordinary adjacent vertebral bodies was 30.14 ± 3.40. But they were significantly smaller than those before operation. It is consistent with the report of Zhou Xs.^[21]^ That the original discomfort disappeared after cement injection into the fractured vertebral body of OVCFs patients. This further confirmed the feasibility of cement strengthening of the fractured vertebral body, as the heat released by cement solidification was sufficient to destroy the nerves causing the patient’s low back pain.^[22]^

Theoretically, the sandwich vertebral body receives double load transmission from the upper and lower vertebral bodies, which is more prone to endplate collapse. But our research has overturned this theory. During the follow-up period, a total of 8 patients had fractures near the cement reinforced vertebral body, 1 (10.00%) occurred in the sandwich vertebral body, that is, the sandwich vertebral body fracture, and the other 7 (3.26%) had ordinary adjacent vertebral fractures. It should be noted that the incidence of sandwich vertebral fractures was 10.00%, which was not statistically higher than 3.26% for ordinary adjacent vertebral fractures. This is consistent with the conclusion of Ping-Yeh et al.^[20]^ Although the incidence of sandwich vertebral body and ordinary adjacent vertebral body fractures in this study is <21.3% and 16.4% in Ping-Yeh study.^[20]^ However, the results are very different from the long-term studies conducted by Liu et al.^[23]^ Liu et al^[23]^ reported that the incidence of sandwich vertebral fracture was 12.9%, which was statistically higher than that of ordinary adjacent vertebral fracture of 6.2%, and they considered that 85% of sandwich vertebral fractures occurred 5 years after operation. The average follow-up time of sandwich vertebral body patients and ordinary adjacent vertebral body patients was 31.30 ± 18.04 months and 25.85 ± 7.96 months, the follow-up time was short, which may be the reason why it is very different from the research conclusions of Liu et al.^[23]^

All OVCFs patients treated with PKP in our medical institution were in the early stage of the main complaint of low back pain. Therefore, cement may have less of an acceleration of adjacent vertebral degeneration, since the callus has not largely formed and the cement intersects with the surrounding fractured trabeculae. In addition, it may be that the strong stress of the cement-augmented vertebral body on the adjacent vertebrae is partially counteracted by the surrounding soft tissue, making osteoporosis, the patient’s underlying disease, a major factor in new vertebral fractures.^[24]^ Finally, our medical practitioners do not strive for a perfect cement-to-bone contact between the upper and lower endplates of fractured vertebrae. This reduces the stress changes in the spinal unit and reduces the risk of recurrent fracture.

The fractured sandwich vertebral body is located in L2, which is around the maximum range of motion of the vertebral body. Faced with a high fracture rate of 10.00% of the sandwich vertebrae, we felt it was necessary to take measures to reduce the risk of refracture. At the beginning of this century, some researchers^[25]^ injected cement into adjacent intact vertebral body after the fractured vertebral body was strengthened with cement, it was found that the subsequent fracture rate of adjacent vertebral bodies was significantly reduced and satisfactory results were achieved. Recently, when Jia et al^[26]^ injected cement into the fractured vertebral body, they also injected cement into the sandwich vertebral body, which is a special adjacent vertebral body. Surprisingly, none of the sandwich vertebrae patients who underwent prophylactic cement injections during follow-up were found to have new fractures. Therefore, we suggest that the sandwich vertebral body can be injected with cement as appropriate to reduce the incidence of subsequent fractures.

This study makes it clear that the incidence of sandwich vertebral fracture is not higher than that of ordinary adjacent vertebral body. But some study limitations still exist in our study. To start with, this was a single-center retrospective study with a small number of cases and a short-term follow-up. Second, patients received standardized antiosteoporosis treatment after operation, which may affect our statistics of fracture incidence in different patients. Finally, the bone mineral density in all patients was not recorded by us. It is also an important thing.

## 5. Conclusion

PKP is a safe and effective treatment for patients with OVCFs. Although the volume of cement in a single vertebral body is less and the procedure time and bleeding are more, the incidence of sandwich vertebral fracture is not higher than that of ordinary adjacent vertebral body.

### Author contributions

Bo Yang conceived the research design, Bo Yang collected data and papered the article, Yangxue Zhao revised this article, Yangxue Zhao is responsible for this article.
